# An HIV elite controller patient carrying the homozygous H63D variant in the homeostatic iron regulator gene: A case report

**DOI:** 10.1097/MD.0000000000027732

**Published:** 2021-11-12

**Authors:** Isabella Zanella, Emanuele Focà, Melania Degli-Antoni, Francesco Castelli, Eugenia Quiros-Roldan

**Affiliations:** a Department of Molecular and Translational Medicine, University of Brescia, Clinical Chemistry Laboratory, Cytogenetics and Molecular Genetics Section, Diagnostic Department, ASST Spedali Civili di Brescia, Brescia, Italy; b University Division of Infectious and Tropical Diseases, University of Brescia and ASST Spedali Civili, Brescia, Italy.

**Keywords:** case report, elite controller, H63D, HFE variant, HIV

## Abstract

**Rationale:**

HIV elite controllers represent a rare subset of persons living with HIV, able to spontaneously control viral replication without antiviral therapy. HLA-B∗57 and HLA-B∗27 alleles are associated to efficient polyfunctional CD8^+^ T-cell response and are overrepresented in elite controllers but these alleles alone incompletely explain spontaneous HIV replication control in these subjects. Further mechanisms involved in innate and adaptive immune response and host genetics may contribute to this control. In this context, the homeostatic iron regulator (HFE) gene encodes a major histocompatibility complex-class-I-like molecule involved in both innate immunity, acting also through autophagy regulation, and iron homeostasis, strictly related to immune functions and susceptibility to infections.

**Patient concerns:**

Homozygousity for the p.His63Asp (H63D) variant in the HFE gene was identified in an 80-year-old HIV-infected woman with spontaneous control of viral replication.

**Diagnosis:**

HIV-1 RNA was undetectable in patient's serum with a routine assay and an ultra-sensitive assay (<1 copy/mL) during the 30 years follow-up. CD4^+^ and CD8^+^ T cell counts were stable and normal during all this period.

**Interventions:**

The patient had a history of absence of any physical ailment and no antiviral therapy has been prescribed during the 30 years of follow-up. The subject did not harbor HLA-B∗57 and HLA-B∗27 alleles. HFE gene was sequenced by Sanger, as part of a larger study on a cohort of HIV infected patients, aged >65 years and screened for polymorphisms in genes belonging to several pathways involved in neuroinflammation.

**Outcomes:**

The woman had CD4^+^ and CD8^+^ T cell normal values and spontaneously controlled serum HIV-1 RNA levels for 30 years.

**Lessons:**

We assume that the interplay between the HFE H63D variant in homozygosity and innate immunity, perhaps through autophagy regulation, could play a role in HIV-1 replication control in our patient. This hypothesis needs to be explored in in vitro and in vivo studies. Understanding mechanisms involved in spontaneous control of HIV-1 replication remains indeed a challenge due to its possible implications for HIV cure research.

## 1 Introduction

A little subset of HIV infected people spontaneously controls viral replication in the absence of antiviral therapy (ART). These individuals, named elite controllers (ECs), represent less than 1% of all HIV-infected patients and are characterized by viral loads undetectable by standard assays, strong HIV specific CD8^+^ T-cell responses and normal CD4^+^ T-cell counts. Great heterogeneity has, however, been described in these individuals regarding residual plasma viremia, extent of viral replication control, duration of stability of CD4^+^ T-cell counts and clinical outcome.^[1]^ Several host factors and virus characteristics seem associated with spontaneous HIV control. In some cases, control of viremia is due to infection with attenuated HIV variants, although host factors may be more relevant even in these cases.^[2]^ Integration sites of proviral sequences seem to be preferentially located in chromosome regions with heterochromatin features in ECs in contrast to individuals treated with long-term ART.^[3]^ The strong HIV-specific CD8^+^ T cells response, the main immunological feature of ECs, is partly due to host genetics.

About 25% to 40% of ECs harbor protective Human Leukocyte Antigen (HLA) class I alleles, like HLA-B∗52, HLA-B∗57, HLA- B∗14, and HLA-B∗27, that have been associated to efficient polyfunctional CD8^+^ T-cell response. However, not all ECs have those protective HLA alleles nevertheless exhibit a strong HIV-specific CD8^+^ T cell response. CD4^+^ T cells, humoral immune response, antibody-dependent functions, and innate immune cells may further contribute to the spontaneous control of HIV-1 infection and the role of sex, ethnicity, and host genetics is still debated.^[4–6]^

## 2 Case presentation

**Figure 1:**
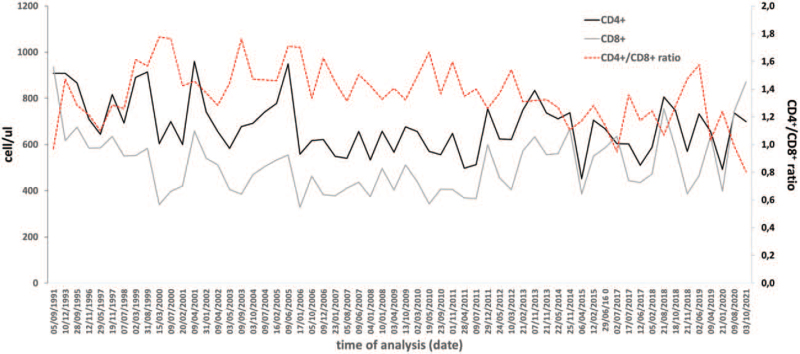
CD4^+^ and CD8^+^ T-cell counts over time during the follow-up of the elite controller case here reported.

Here we present the case of an 80-year-old woman, first tested for HIV in 1990 when she was 50 years old and regularly followed until July 2021. Western-blot showed reactivity for gp160, gp120, p65, p55, gp41, p40, p32, p24, and p18 of HIV-1 and no reactivity for HIV-2 proteins. Plasmatic HIV-1 RNA was undetectable with our routine assay (<20 copies/mL) during all the follow-up. At her last visit, hypertension, non-insulin-dependent diabetes, and mild kidney disease were present comorbidities while both neurocognitive and motorial test were normal according to her age. HIV-1 RNA was not detected in serum with an ultra-sensitive assay (<1 copy/mL) in 1999, 2003, and 2015, while total HIV-1 DNA was detected in peripheral blood mononuclear cells (PBMCs) collected in 2010. Patient's CD4^+^ and CD8^+^ T-cells and CD4^+^/CD8^+^ remained normal throughout the follow-up (Fig. 1). HLA testing revealed HLA-B∗44 and B∗51 positivity. No ART was prescribed during the follow-up.

This patient belongs to a cohort of HIV infected patients, aged >65 years and screened in our department for neurocognitive impairment, including search for gene polymorphisms belonging to several pathways involved in neuroinflammation like iron metabolism and lipid homeostasis. The study was approved by the Ethics Committee of ASST-Spedali Civili di Brescia and informed consent for genetic screening was obtained. Genomic DNA was extracted from a peripheral blood sample using the Wizard Genomic DNA Purification kit (Promega Corporation, Madison, WI). Extracted DNA was quantified by the use of Qubit 2.0 Fluorometer (Thermo Fisher Scientific, Waltham, MA), with Qubit dsDNA HS Assay Kit (Thermo Fisher Scientific). Genomic DNA was amplified by polymerase chain reaction, with previously described primers and conditions.^[7]^ Amplicons were analyzed by direct Sanger sequencing on ABI 3500 Genetic Analyzer (Thermo Fisher Scientific). Sequencing results were analyzed using Chromas software. Within the cohort, we found that the only EC also was the only patient homozygous for the p.His63Asp (H63D) variant in the homeostatic iron regulator (HFE) gene, one of the main gene involved in human hereditary hemochromatosis (HH). Iron homeostasis biomarkers of patient were however normal.

## 3 Discussion

The HFE gene encodes a major histocompatibility complex (MHC)-I-like molecule involved in both iron homeostasis and immunity. HFE is a transmembrane protein, exposed with β-2 microglobulin on the cell surface of many cells, particularly hepatocytes, Kupffer cells, monocytes, macrophages, granulocytes, and intestinal cells. It is composed of 3 extracellular α domains, a transmembrane domain, and a cytoplasmatic tail. Binding with β-2 microglobulin occurs through the α3 domain and is required for cell surface expression, like for classical MHC-I molecules. α1/α2 domains are mainly responsible for HFE interaction with the transferrin receptor 1, the main cellular receptor involved in iron uptake from the extracellular milieu through the binding with transferrin (Tf), a blood protein that binds circulating iron.

Since the discovery of the involvement of HFE gene mutations in HH, the role of HFE in iron homeostasis has been thoroughly characterized.^[8]^ HFE regulates cellular iron uptake but also senses blood iron levels and regulates expression of the major regulator of systemic iron homeostasis, the peptide hepcidin. The molecular mechanisms involved in this regulation are not yet completely understood. Hepcidin, released in the circulation, acts inducing the degradation of the cellular iron exporter ferroportin both in the basolateral surface of enterocytes, reducing intestinal iron absorption, and in macrophages, reducing iron recycling and resulting in intracellular iron accumulation, in turn reducing blood iron level and Tf saturation.^[8]^

In all cell types, classical MHC-I molecules are involved in adaptive immunity through the displaying of antigens that are processed within the cells, like invading or intracellular pathogens, to CD8^+^ cytotoxic T-cells. MHC-I molecules present antigens to these cells through the interaction of T-cell receptor with the open groove in their α1/α2 domains, while the α3 domain engages the CD8 molecule. Non-classical MHC-I molecules (i.e., HFE) are both involved in adaptive and innate immunity and engaged in the regulation of T- and natural killer cells. HFE, although does not bind peptides, is recognized by the T-cell receptor of a specific subset of CD8^+^ T-cells in mice and is able of shaping the T-cell repertoire.^[9–11]^ Moreover, HFE, through its α1/α2 domains, seems to be a negative regulator of CD8^+^ T-cell activation, reducing the release of several soluble factors like macrophage inflammatory protein-1β and interferon (IFN)-ϒ and reducing the expression of markers of T-cell activation: this could have clear implications in the immune response to pathogens and autoimmune diseases.^[12]^

HFE-related HH is characterized by low hepcidin production, elevated serum Tf saturation, serum ferritin levels, and iron overload in tissue and organs like liver, skin, pancreas, and heart. HH is mainly associated with homozygosity for the p.Cys282Tyr (C282Y) variant, present in 0.4% of Caucasian people. Homozygosity for the C282Y variant has however a low penetrance. C282Y/H63D compound heterozygotes have lower risk of developing iron overload than C282Y homozygotes and only some individuals who are heterozygous for either C282Y or H63D have elevated serum Tf saturation and ferritin levels, but they do not develop complications of iron overload. People homozygous for the H63D allele, like the EC subject here reported, are relatively rare (1%–2% of the general population) and are considered at very low risk of HH.

Patients with HH due to homozygosity for C282Y variant are also affected by several immune defects like low number of CD8^+^ T-lymphocytes affecting CD4^+^/CD8^+^ ratios, lower levels of MHC-I expression in PBMCs due to an increased rate of MHC-I endocytosis and increased occurrence of autoimmune diseases.^[9]^ Nonetheless, HFE C282Y mutation may offer an advantage to asymptomatic patients, interfering with the inflammatory response of macrophages. Reduced iron levels in macrophages of C282Y allele carriers make them less susceptible to bacteria that depend on macrophage iron like *Salmonella Typhimurium*, *Mycobacterium tuberculosis*, *Leishmania amazonensis*, *Chlamydia* or *Legionella* species. This advantage against some infectious diseases may explain the high frequency of HFE mutations in the Caucasian population.^[9]^

People homozygous for the H63D allele, like the EC subject here reported, are relatively rare (1%–2% of the general population) and are considered at very low risk of HH.^[13]^

Little is known about the effects of the HFE H63D variant on the immune system but some interesting findings are described in the literature. The H63D variant, located in the α1 domain of HFE protein, retains only partially the negative regulation of CD8^+^ T-cell activation observed for wild type HFE.^[12]^ Further, mutated transcripts predominate on the wild-type transcripts and mutated protein is increased in PBMCs from H63D heterozygotes.^[14]^ Iron is strictly involved in macrophage functions and C282Y homozygotes have limited iron storage in macrophages despite iron overload in several tissues. Wild-type HFE leads to iron accumulation in the human macrophage THP-1 cell line through the inhibition of iron release, while H63D mutated HFE abolish this inhibitory effect.^[15]^ Murine macrophages harboring the H67D variant, corresponding to the human H63D, show lower cellular proliferation under iron exposure, higher L- ferritin expression suggestive of higher iron storage capability, reduced cell migration, higher phagocytic activity, and altered cytokine release profile suggestive of decreased inflammatory response compared with the wild-type counterpart.^[16]^ H67D mice have increased survival over wild-type mice after experimental cerebral malaria due to the infection with Plasmodium falciparum.^[17]^ Finally, the H63D mutation has been associated with long-term virus suppression in chronic hepatitis C patients treated with IFN-based therapy in a large meta-analysis.^[18]^ All together these findings suggest that H63D variant may have an impact and, in some cases, also positively influence the immune response against some pathogens.

Furthermore, autophagy has recently been described as increased in cells carrying the H63D variant, probably through the inhibition of the mammalian target of rapamycin complex 1 (mTORC1), a negative regulator of autophagy.^[19]^ Autophagy is a process of degradation and recycling of cellular components and invading pathogens, involved in cellular homeostasis and activation of innate and adaptive immunity, through the regulation of inflammatory responses and antigen presentation. Interestingly, HFE has been recently identified as a negative regulator of antiviral innate immunity related to type I IFNs, acting on the degradation of antiviral signaling proteins through selective autophagy.^[20]^ Type I IFNs are important mediators in the control of HIV infection.^[21]^ These findings also evidence the strict cross-talk among iron balance, autophagy, and innate immune response.^[20]^ Autophagy also plays an important role in HIV-1 replication,^[22]^ especially in ECs.^[23,24]^ The treatment of PBMCs isolated from ECs with rapamycin, an inhibitor of mTORC1, promotes autophagy, resulting in the impairment of viral production.^[23]^ Further, in humanized mouse models pan-inhibitors of mTORC1 inhibit HIV-1 infection by interfering with virus entry, through the reduction of C-C chemokine receptor type 5 levels, and with the transcription of HIV genes.^[25]^

Considering all the above findings, our hypothesis is that the interplay between H63D mutant HFE and innate immunity, perhaps through autophagy regulation, could play a role in HIV-1 replication control in our patient. Certainly, it will be necessary to screen more ECs patients for HFE mutations and explore our hypothesis with in vitro and in vivo studies, but we think that it may be an interesting hypothesis for future research. Understanding mechanisms involved in spontaneous control of HIV-1 replication remains indeed a challenge due to its possible implications for HIV cure research.

## Author contributions

**Conceptualization:** Isabella Zanella, Eugenia Quiros-Roldan.

**Data curation:** Isabella Zanella, Eugenia Quiros-Roldan.

**Investigation:** Isabella Zanella, Eugenia Quiros-Roldan.

**Methodology:** Isabella Zanella.

**Supervision:** Isabella Zanella, Eugenia Quiros-Roldan.

**Validation:** Emanuele Focà.

**Visualization:** Emanuele Focà, Melania Degli-Antoni, Francesco Castelli, Eugenia Quiros-Roldan.

**Writing – original draft:** Isabella Zanella, Eugenia Quiros-Roldan.

**Writing – review & editing:** Isabella Zanella, Emanuele Focà, Melania Degli-Antoni, Francesco Castelli, Eugenia Quiros-Roldan.

## References

[1] Navarrete-Muñoz MA Restrepo C Benito JM Rallón N. Elite controllers: a heterogeneous group of HIV-infected patients. Virulence 2020;11:889–97. 32698654 10.1080/21505594.2020.1788887 PMC7549999

[2] Zaunders J Dyer WB Churchill M. The Sydney Blood Bank Cohort: implications for viral fitness as a cause of elite control. Curr Opin HIV AIDS 2011;6:151–6. 21378562 10.1097/COH.0b013e3283454d5b

[3] Jiang C Lian X Gao C. Distinct viral reservoirs in individuals with spontaneous control of HIV-1. Nature 2020;585:261–7. 32848246 10.1038/s41586-020-2651-8 PMC7837306

[4] International HIV Controllers Study. The major genetic determinants of HIV-1 control affect HLA class I peptide presentation. Science 2010;330:1551–7. 21051598 10.1126/science.1195271 PMC3235490

[5] Almeida JR Price DA Papagno L. Superior control of HIV-1 replication by CD8+ T cells is reflected by their avidity, polyfunctionality, and clonal turnover. J Exp Med 2007;204:2473–85. 17893201 10.1084/jem.20070784 PMC2118466

[6] Gonzalo-Gil E Ikediobi U Sutton RE. Mechanisms of virologic control and clinical characteristics of HIV+ elite/viremic controllers. Yale J Biol Med 2017;90:245–59. 28656011 PMC5482301

[7] Gazzina S Premi E Zanella I. Iron in frontotemporal lobar degeneration: a new subcortical pathological pathway? Neurodegener Dis 2016;16:172–8. 26613252 10.1159/000440843

[8] Barton JC Edwards CQ Acton RT. HFE gene: structure, function, mutations, and associated iron abnormalities. Gene 2015;574:179–92. 26456104 10.1016/j.gene.2015.10.009 PMC6660136

[9] Reuben A Chung JW Lapointe R Santos MM. The hemochromatosis protein HFE 20 years later: an emerging role in antigen presentation and in the immune system. Immun Inflamm Dis 2017;5:218–32. 28474781 10.1002/iid3.158 PMC5569368

[10] Rohrlich PS Fazilleau N Ginhoux F. Direct recognition by alphabeta cytolytic T cells of Hfe, a MHC class Ib molecule without antigen-presenting function. Proc Natl Acad Sci U S A 2005;102:12855–60. 16123136 10.1073/pnas.0502309102 PMC1200262

[11] Cardoso C Porto G Lacerda R. T-cell receptor repertoire in hereditary hemochromatosis: a study of 32 hemochromatosis patients and 274 healthy subjects. Hum Immunol 2001;62:488–99. 11334672 10.1016/s0198-8859(01)00233-6

[12] Reuben A Phénix M Santos MM Lapointe R. The WT hemochromatosis protein HFE inhibits CD8+ T-lymphocyte activation. Eur J Immunol 2014;44:1604–14. 24643698 10.1002/eji.201343955

[13] Anderson GJ Bardou-Jacquet E. Revisiting hemochromatosis: genetic vs. phenotypic manifestations. Ann Transl Med 2021;9:731 doi: 10.21037/atm-20-5512. 33987429 10.21037/atm-20-5512 PMC8106074

[14] Rosmorduc O Poupon R Nion I. Differential HFE allele expression in hemochromatosis heterozygotes. Gastroenterology 2000;119:1075–86. 11040194 10.1053/gast.2000.18146

[15] Drakesmith H Sweetland E Schimanski L. The hemochromatosis protein HFE inhibits iron export from macrophages. Proc Natl Acad Sci U S A 2002;99:15602–7. 12429850 10.1073/pnas.242614699 PMC137763

[16] Nixon AM Neely E Simpson IA Connor JR. The role of HFE genotype in macrophage phenotype. J Neuroinflammation 2018;15:30 doi: 10.1186/s12974-018-1057-0. 29391061 10.1186/s12974-018-1057-0 PMC5796391

[17] Leitner DF Stoute JA Landmesser M Neely E Connor JR. The HFE genotype and a formulated diet controlling for iron status attenuate experimental cerebral malaria in mice. Int J Parasitol 2015;45:797–808. 26296689 10.1016/j.ijpara.2015.07.003

[18] Li SH Zhao H Ren YY. The H63D mutation of the hemochromatosis gene is associated with sustained virological response in chronic hepatitis C patients treated with interferon-based therapy: a meta-analysis. Tohoku J Exp Med 2012;226:293–9. 22499121 10.1620/tjem.226.293

[19] Kim Y Stahl MC Huang X Connor JR. H63D variant of the homeostatic iron regulator (HFE) gene alters α-synuclein expression, aggregation, and toxicity. J Neurochem 2020;155:177–90. 32574378 10.1111/jnc.15107

[20] Liu J Wu X Wang H. HFE inhibits type I IFNs signaling by targeting the SQSTM1-mediated MAVS autophagic degradation. Autophagy 2020;17:01–16. 10.1080/15548627.2020.1804683 PMC8386699 32746697

[21] Bourke NM Napoletano S Bannan C. Control of HIV infection by IFN-α: implications for latency and a cure. Cell Mol Life Sci 2018;75:775–83. 28988399 10.1007/s00018-017-2652-4 PMC11105398

[22] Cabrera-Rodríguez R Pérez-Yanes S Estévez-Herrera J. The interplay of HIV and autophagy in early infection. Front Microbiol 2021;12:661446 doi: 10.3389/fmicb.2021.661446. 33995324 10.3389/fmicb.2021.661446 PMC8113651

[23] Ciccosanti F Corazzari M Casetti R. High levels of TRIM5α are associated with xenophagy in HIV-1-infected long-term nonprogressors. Cells 2021;10:1207 doi: 10.3390/cells10051207. 34069225 10.3390/cells10051207 PMC8156091

[24] Loucif H Dagenais-Lussier X Beji C. Lipophagy confers a key metabolic advantage that ensures protective CD8A T-cell responses against HIV-1. Autophagy 2021;01–16. 10.1080/15548627.2021.1874134 PMC8632342 33459125

[25] Heredia A Le N Gartenhaus RB Sausville E. Targeting of mTOR catalytic site inhibits multiple steps of the HIV-1 lifecycle and suppresses HIV-1 viremia in humanized mice. Proc Natl Acad Sci U S A 2015;112:9412–7. 26170311 10.1073/pnas.1511144112 PMC4522811

